# Prioritizing investments in innovations to protect women from the leading causes of maternal death

**DOI:** 10.1186/1471-2393-14-10

**Published:** 2014-01-09

**Authors:** Tara M Herrick, Claudia M Harner-Jay, Alice M Levisay, Patricia S Coffey, Michael J Free, Paul D LaBarre

**Affiliations:** 1 PATH, PO Box 900922, Seattle, WA 98109, USA

**Keywords:** Maternal health, Postpartum hemorrhage, Preeclampsia, Eclampsia, Technology assessment, Oxytocin, Magnesium sulfate, Uterine balloon tamponade, Blood pressure measurement, Proteinuria test

## Abstract

PATH, an international nonprofit organization, assessed nearly 40 technologies for their potential to reduce maternal mortality from postpartum hemorrhage and preeclampsia and eclampsia in low-resource settings. The evaluation used a new Excel-based prioritization tool covering 22 criteria developed by PATH, the Maternal and Neonatal Directed Assessment of Technology (MANDATE) model, and consultations with experts. It identified five innovations with especially high potential: technologies to improve use of oxytocin, a uterine balloon tamponade, simplified dosing of magnesium sulfate, an improved proteinuria test, and better blood pressure measurement devices. Investments are needed to realize the potential of these technologies to reduce mortality.

## Background

Innovations in health technologies and services are often least available to people with the greatest health needs [1,2]. Because many women in low-resource settings lack access to basic maternal health technologies and services, more than 30 women die each hour from complications related to pregnancy and childbirth, mostly in sub-Saharan Africa and South Asia [3,4].

Many factors make it difficult to get needed maternal health innovations to women in low-resource settings [1]. These include serious health system deficiencies, such as the limited capacity of health staff. They also include technology-focused challenges, such as:

• Complicated user requirements for technologies, especially for health workers without advanced training.

• Weak or nonexistent distribution systems.

• Disparate markets and small sales volumes that drive up costs for manufacturing and distribution.

• Expensive clinical studies.

• Restrictive policy and regulatory guidelines.

Targeted investment and collaboration to address these challenges may help to bring lifesaving technologies to all women rather than just those in the richest countries.

Millennium Development Goal 5 aims to reduce the maternal mortality ratio by 75 percent by 2015 [3,4]. To accelerate progress toward this goal, Merck & Co., Inc., launched *Merck for Mothers*, a ten-year initiative to reduce maternal mortality.

In October 2011, PATH received funding through *Merck for Mothers* to identify the most promising technologies with the potential to help save women’s lives. PATH is an international nonprofit organization with nearly four decades of experience adapting, developing, and introducing health technologies for developing countries.

## Methods

PATH identified 38 technologies for evaluation, with emphasis on those that address postpartum hemorrhage and preeclampsia and eclampsia, the leading causes of maternal death. We identified these technologies by reviewing World Health Organization (WHO) guidelines, Bill & Melinda Gates Foundation Grand Challenges in Global Health awards, and US Agency for International Development Saving Lives at Birth finalists, as well as by soliciting input from maternal health experts and other stakeholders.

We used a rigorous, multiphased approach to evaluate the technologies and then create strategies to advance those with the greatest promise. The goal of phase 1 was to identify the 10 best technology opportunities for additional analysis. To winnow the list, PATH needed a way to assess all the criteria that affect a technology’s potential impact.

Because our review of the literature found that no existing assessment tool would meet our needs, PATH developed a strategic prioritization tool for the project [see Additional file 1]. The tool is a decision matrix consisting of 22 criteria, each representing a discrete element of the technology’s value proposition and potential for impact (see Table 1). The matrix captures a score for each criterion, a cumulative score, and score substantiation for each technology. To develop the criteria and related definitions, project staff consulted internal maternal health experts, commercial firms, and other sources [5]. The tool and detailed information on the criteria, definitions, and process are available online at http://sites.path.org/mnhtech/assessment/tool/. (If the Uniform Resource Locator [URL], or web address, for a site mentioned in this manuscript changes, the authors will inform readers by adding a comment to the manuscript online).

**Table 1:** Assessment criteria for technologies that address postpartum hemorrhage and preeclampsia and eclampsia

**Category**	**Criteria**	Evaluation approach (high, medium, low)*
Gap	Gap-filling potential for health	Evaluate what layers of the treatment continuum could most greatly reduce mortality (e.g., prevention, diagnostics, treatment), the strength of the available data, and the percentage of cases that could be managed
Technology performance	Clinical evidence (efficacy/effectiveness)	Superior to benchmark,** similar to benchmark, inferior to benchmark
	Safety (patient/health care worker)	Superior to benchmark,** similar to benchmark, inferior to benchmark
	Ease of use	Superior to benchmark,** similar to benchmark, inferior to benchmark
	Usage requirements (e.g., durability, shelf life, electricity, storage temperature)	Technology has two or fewer usage requirements, technology has between three and five usage requirements, technology has greater than five usage requirements
Enabling factors	Alignment with internationally recognized guidelines (e.g., the World Health Organization, the International Federation of Gynecology and Obstetrics, and the International Confederation of Midwives)	Technology is recommended by at least one organization, divergent opinions exist, technology is not recommended
	Donor financial support (product development or implementation)	Funding greater than US$5 M, between US$1 M and US$5 M, less than US$1 M (or no funding identified)
	Other nonfinancial support	Placeholder for other nonfinancial supporters that may not be captured elsewhere; not scored
	Acceptability profile	Broadly acceptable, mixed, acceptable in few geographies (South Asia and sub-Saharan Africa)
	Organizational capabilities	All key partners have experience with the technology, a subset of key partners has experience with the technology, none of the key partners has experience with the technology
Market analysis	Manufacturing costs	Superior to benchmark** (lower cost), similar to benchmark, inferior to benchmark (higher cost)
	Distribution system requirements (warehouse, cold chain, transportation factors)	Infrastructure for a distribution channel(s) exists and high likelihood of utilization, infrastructure for distribution channel(s) exists and moderate likelihood of utilization, distribution channel(s) does not exist or a low likelihood of utilization
	Manufacturing plan established	Capture manufacturing information including delays, hurdles, risk, and complexity; not scored
	Target setting (community, primary health care, hospital)	Capture setting(s) where the technology would be used; not scored
	Target provider/administrator	Community health care worker, nurse or midwife, physician
	Potential multiple markets (additional value to the health care system)	Additional uses identified (high), no additional uses (low)
	Technology readiness level (clinical/regulatory development)	Regulatory or commercialization, confirmatory, discovery, or exploratory
	Cost of clinical development	Less than US$5 M, between US$5 M and US$50 M, greater than US$50 M
	Clarity of regulatory/clinical pathways	Class I US Food and Drug Administration (USFDA) device, class II USFDA device, class III USFDA device or requires efficacy data (e.g., drugs)
Unique considerations	System requirements (disruption)	Low system requirements (training/infrastructure), modest system requirements, high system requirements
	Product bundling	No other technologies required for impact, likely need to bundle with one technology, likely need to bundle with two or more technologies
	Other	Placeholder for other unique considerations; not scored

^*^Detailed definitions are available at http://sites.path.org/mnhtech/assessment/tool/.

^**^Benchmark based on WHO treatment guidelines or the best benchmark for that technology category.

The tool encompasses five categories of criteria that influence potential impact:

• *The gap-filling potential for health*. This category focuses on the technology’s potential for reducing morbidity and mortality and filling existing “gaps.” Technologies that are used earlier in the treatment continuum (e.g., prevention) generally receive a higher ranking because of their potential to prevent life-threatening conditions, reduce unnecessary morbidity and mortality, and reduce the need for emergency care. Other considerations in this category include the strength of available data and the percentage of cases that could be better managed with the technology.

• *Technology performance.* The criteria in this category concern the clinical performance, safety, and ease of use of the technology compared to an existing benchmark, which we identified by reviewing WHO guidelines. We also evaluated usage requirements to highlight factors that may influence appropriate use in low-resource settings. These requirements include fragile handling, shelf life, waste disposal, specialized storage needs, electrical power requirements, infection prevention, and training.

• *Enabling factors.* We use this category to determine whether an enabling environment is in place for successful technology introduction. Factors include alignment with international guidelines, existing support, an acceptability profile, and existing capabilities.

• *Market considerations*. We use the criteria in this category to evaluate the time and investment needed to bring a technology to market as well as the health care setting and type of health worker most likely to use the technology.

• *Unique considerations*. With this category, we assess other factors that may significantly affect the introduction and scaling of the technology, such as system and bundling requirements and potential disruption of existing systems. “Disruption” is defined as any significant shift in costs, roles, or risks of any of the stakeholders that are involved in the acquisition, distribution, or deployment of technologies.

In phase 2 of the assessment, PATH applied different methods to further narrow the focus to a handful of technologies with the greatest promise. For example, PATH used the Maternal and Neonatal Directed Assessment of Technology (MANDATE) model developed by Research Triangle Institute to assess the potential number of lives saved for each technology (for more information, see http://mnhtech.org). We also conducted an online survey with maternal health experts to rank candidate technologies for their potential to reduce mortality in sub-Saharan African and South Asia. The survey was sent to 63 maternal health experts with developing-country expertise from a variety of organizations and was completed by 34 individuals (54% response rate) in April 2012.

## Results and discussion

Our systematic evaluation identified five types of technologies with especially high potential for reducing deaths from postpartum hemorrhage and preeclampsia and eclampsia in low-resource settings:

• *Technologies to facilitate appropriate use of oxytocin*. Although WHO recommends oxytocin for preventing and managing postpartum hemorrhage, this drug is unavailable to many women, as highlighted in a recent report from the United Nations Commission on Life-Saving Commodities for Women and Children [6]. Expanding its use will likely require developing and introducing easy-to-use formats appropriate for low-resource settings. Developing heat-stable formulations, for example, may eliminate or reduce the need for cold chain storage and enable broader use, especially for home births [7]. Also, formulations for alternative methods of administration (e.g., via inhalation or sublingual delivery) may increase safety and allow even lower-level health workers to give the medicine. There is a need for accelerated investment in new heat-stable formulations and alternative administration methods.

• *Uterine balloon tamponade*. This elastomeric bladder-like device can be inserted into the uterus and filled with saline to control severe postpartum bleeding [8]. It works rapidly and effectively, reducing the need for risky surgical interventions and blood transfusions [9,10]. Commercially available models, however, are too expensive for widespread use in developing countries, and performance of some versions is inconsistent. Investments are needed to gather clinical evidence for efficacy in low-resource settings through randomized, controlled trials of new, affordable devices under development.

• *Simplified dosing of magnesium sulfate.* WHO has identified magnesium sulfate as the most effective, safe, and low-cost medication for preventing and treating convulsions for women with severe preeclampsia or eclampsia [11]. Recommended dosing, however, requires a complicated combination of intravenous and intramuscular injection, and health workers in peripheral facilities may be unable to provide this treatment. Simplified dosing and dose packaging are feasible and could help providers more easily give women the medicine they need [12]. There is an immediate need for a randomized, controlled trial to determine whether a simplified, single, loading-dose-only regimen can effectively prevent and treat preeclampsia and eclampsia.

• *Improved proteinuria test*. Although detection of proteinuria is critical for diagnosing preeclampsia, most women in developing countries are not tested for proteinuria because tests are either unavailable or simply not used, and existing technologies have poor performance [13]. Investments are needed to assess novel, easy-to-use methods of proteinuria detection in the laboratory and then develop advanced prototypes for evaluation in low-resource settings.

• *Blood pressure measurement devices*. Like testing for proteinuria, measurement of blood pressure is critical for diagnosing preeclampsia. Health care providers in low-resource settings often fail to detect hypertension because suitable measurement devices are not available or they do not have needed skills [14]. There is a need for low-cost blood pressure measurement devices that are validated for use with pregnant women and that conform to WHO recommendations and international standards.

In phase 3 of the project, PATH created strategies to advance select technologies for introduction, scale-up, and ultimately impact.

## Conclusion

Further development and introduction of maternal health technologies designed for use in low-resource settings will help to save many women’s lives. Through a systematic assessment, PATH has identified five technologies with high potential for impact and provided a basis for prioritizing investments. Especially in times of significant resource constraints, it is critical for donors, national governments, businesses, and other stakeholders to determine the best use of scarce resources to improve maternal health. Investing in high-impact technologies and services and collaborating to advance their use will build brighter futures for women, families, communities, and nations.

## Abbreviations

MANDATE: Maternal and neonatal directed assessment of technology; PATH: Program for Appropriate Technology in Health; WHO: World Health Organization.

## Competing interests

The authors declare that they have no competing interests.

## Authors’ contributions

All authors contributed substantially to the assessment underlying this article and to developing, reviewing, and editing the manuscript. All authors approved the final version.

## Pre-publication history

The pre-publication history for this paper can be accessed here:

http://www.biomedcentral.com/1471-2393/14/10/prepub

## Supplementary Material

Additional file 1 Strategic prioritization tool. Click here for file
